# Fine regulation of ARF17 for anther development and pollen formation

**DOI:** 10.1186/s12870-017-1185-1

**Published:** 2017-12-19

**Authors:** Bo Wang, Jing-Shi Xue, Ya-Hui Yu, Si-Qi Liu, Jia-Xin Zhang, Xiao-Zhen Yao, Zhi-Xue Liu, Xiao-Feng Xu, Zhong-Nan Yang

**Affiliations:** 1 Department of Molecular and Cell Biology, School of Life Science and Technology, Tongji University, Shanghai, 200092 China; 2 College of Life and Environmental Sciences, Shanghai Normal University, Shanghai, 200234 China

**Keywords:** Anther, Tapetum, Male sterility, ARF17, 5mARF17

## Abstract

**Background:**

In *Arabidopsis*, the tapetum and microsporocytes are critical for pollen formation. Previous studies have shown that *ARF17* is expressed in microsporocytes and tetrads and directly regulates tetrad wall synthesis for pollen formation. *ARF17* is the direct target of *miR160*, and *promoterARF17*::*5mARF17* (*5mARF17*/WT) transgenic plants, which have five silent mutations within the *miR160*-complementary domain, are sterile.

**Results:**

Here, we found that ARF17 is also expressed in the tapetum, which was defective in *arf17* mutants. Compared with *arf17* mutants, *5mARF17/*WT plants had abnormal tapetal cells and tetrads but were less vacuolated in the tapetum. Immunocytochemical assays showed that the ARF17 protein over-accumulated in tapetum, microsporocytes and tetrads of *5mARF17*/WT plants at early anther stages, but its expression pattern was not affected during anther development. *5mARF17* driven by its native promoter did not rescue the *arf17* male-sterile phenotype. The expression of *5mARF17* driven by the tapetum-specific promoter *A9* led to a defective tapetum and male sterility in transgenic plants. These results suggest that the overexpression of ARF17 in the tapetum and microsporocytes of *5mARF17*/WT plants leads to male sterility. Microarray data revealed that an abundance of genes involved in transcription and translation are ectopically expressed in *5mARF17/*WT plants.

**Conclusions:**

Our work shows that ARF17 plays an essential role in anther development and pollen formation, and ARF17 expression under *miR160* regulation is critical for its function during anther development.

**Electronic supplementary material:**

The online version of this article (10.1186/s12870-017-1185-1) contains supplementary material, which is available to authorized users.

## Background

In flowering plants, male reproductive processes occur in the stamen. After meiosis, haploid microspores further develop into pollen grains within the locules. Each locule contains four somatic layers that surround microsporocytes/microspores/pollen [1]. The pollen wall is typically composed of an exine and intine. The innermost layer of locule sporophytic tissue, the tapetum, is in direct contact with the microspores to provide necessary nutrition and pollen wall materials for pollen grain maturation [2, 3]. The deposition pattern of the exine is determined by callose formation during the tetrad stage [4, 5].

Auxin plays an important role in plant male reproductive development [6, 7]. Both auxin biosynthesis and transportation are involved in pollen development [8–11]. Auxin response factors (ARFs) respond to auxin; ARFs can be targeted to auxin response elements of downstream genes and can function as transcriptional activators or repressors [12, 13]. Several ARFs are involved in anther development and pollen formation. *ARF1* and *ARF2* regulate flowering time and dehiscence [14], and *ARF6* and *ARF8* facilitate gynoecium and stamen development [15, 16]. Loss of function of *ARF17* directly affects the expression of *CALLOSE SYNTHASE 5* (*CALS5*), which is critical for callose synthesis. *arf17* mutants show a thin callose wall and an abnormal exine pattern, resulting in male sterility [4, 17].

MicroRNAs (miRNAs) are 21 nucleotides long and are negative regulators of gene expression in both plants and animals [18]. Most plant miRNAs have the function of cleaving target mRNAs that have a perfect or nearly perfect complementary sequence [19]. In *Arabidopsis*, *miR167* regulates *ARF6* and *ARF8* expression for ovule and anther development [15]. *miR160* is complementary to the transcripts of *ARF10*, *ARF16* and *ARF17.* Changing 5 bases of the *miR160* recognition site without altering the amino acid sequence of the ARF17 protein (*5mARF17*) prevents *miR160*-directed *ARF17* cleavage [20]. *PromoterARF17::5mARF17* transgenic plants (*5mARF17/*WT) are sterile [20]*.* However, the details and mechanism of the effects of *5mARF17* on plant fertility are not clear.

Here, we show that ARF17 is important not only for microsporocyte/tetrad development but also for tapetum development. ARF17 protein was over-accumulated in the tapetum and microsporocytes, and this over-accumulation led to defects in *5mARF17/*WT plants. Therefore, fine regulation of ARF17 is critical for tapetum development and pollen wall formation.

## Results

### ARF17 is essential for tapetum development

**Fig. 1:**
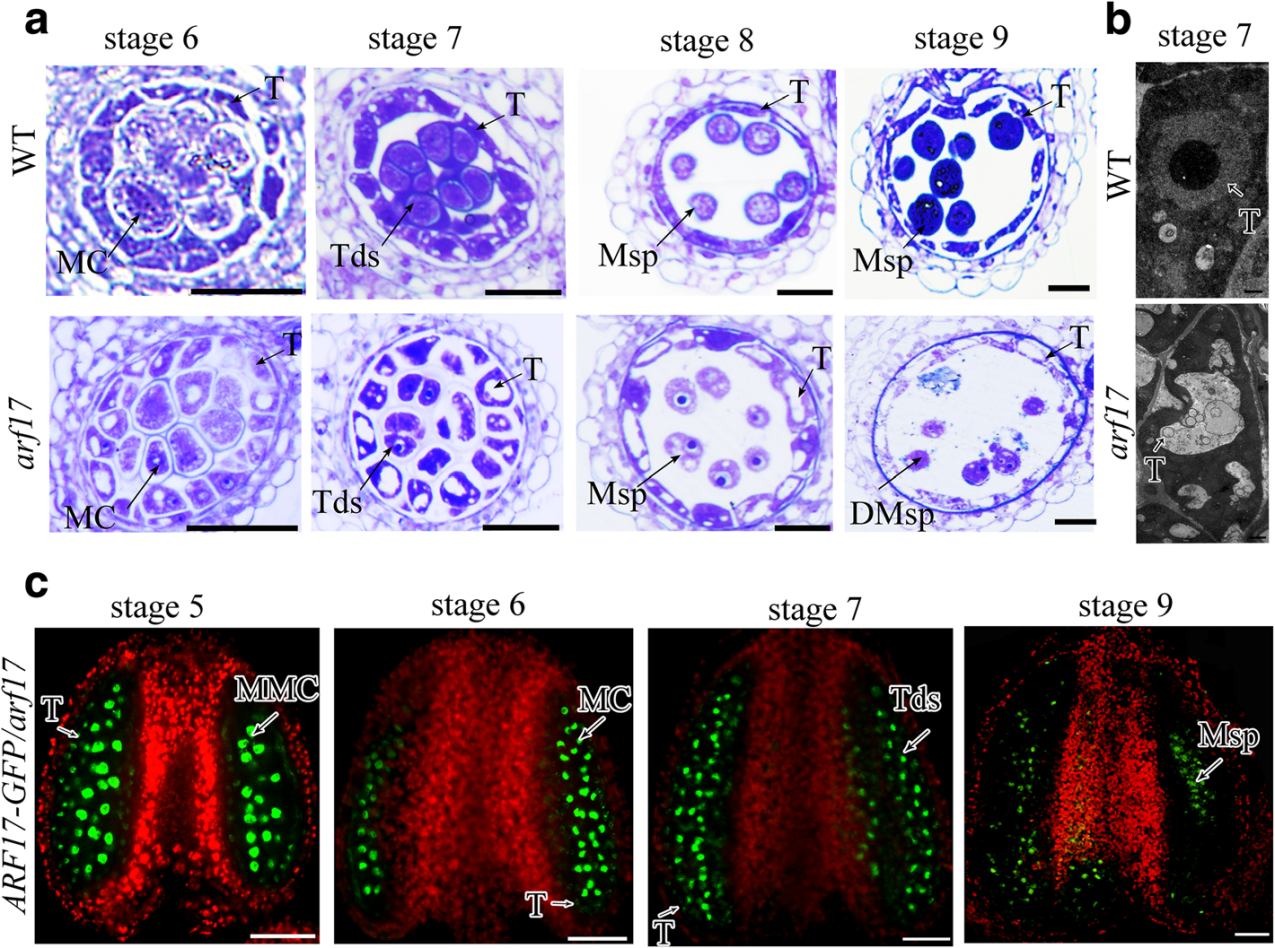
ARF17 is important for tapetum development. **a** Semi-thin sections of the anthers of WT and *arf17* mutant plants. Bar = 20 μm. **b** TEM showing the tapetum morphology of WT and *arf17* plants at anther stage 7. Bar = 500 nm. **c** Fluorescence confocal images indicating GFP signals in the anthers of an *ARF17-GFP/arf17* plant. Bar = 50 μm. Green is the GFP signal (530 nm), and red is chlorophyll autofluorescence. DMsp, degenerated microspore; MC, meiotic cell; MMC, microspore mother cell; Msp, microspore; T, tapetum; Tds, tetrads

Previous investigations have shown that ARF17 directly regulates the expression of *CALS5* for tetrad wall formation. In *arf17* mutants, tetrad walls are much thinner than those of wild type (WT), and the pollen wall pattern is defective [17]. In this work, we found that tapetum development was also defective in *arf17* mutants and was abnormally vacuolated during development from stage 7 to stage 9 (Fig. 1a, b). Previous investigations have shown that the ARF17-GFP signal is localized in microsporocytes, microspores and mature pollen instead of tapetal cells [17]. Here, we rescanned ARF17-GFP signals using confocal laser scanning microscopy in *pARF17::ARF17-GFP/arf17* complementary plants (T_3_ generation) in which ARF17-GFP fully restored the *arf17* male-sterile phenotype. The ARF17-GFP signal was detected in tapetal cells at stage 5, but then the signal weakened from stage 6 to 7 (Fig. 1c). The localization of ARF17-GFP in tapetal cells was consistent with the defective tapetum in the *arf17* mutants. These results suggest that *ARF17* is also essential for tapetum development.

### *5mARF17*/WT plants are defective in both the tapetum and microsporocytes

**Fig. 2:**
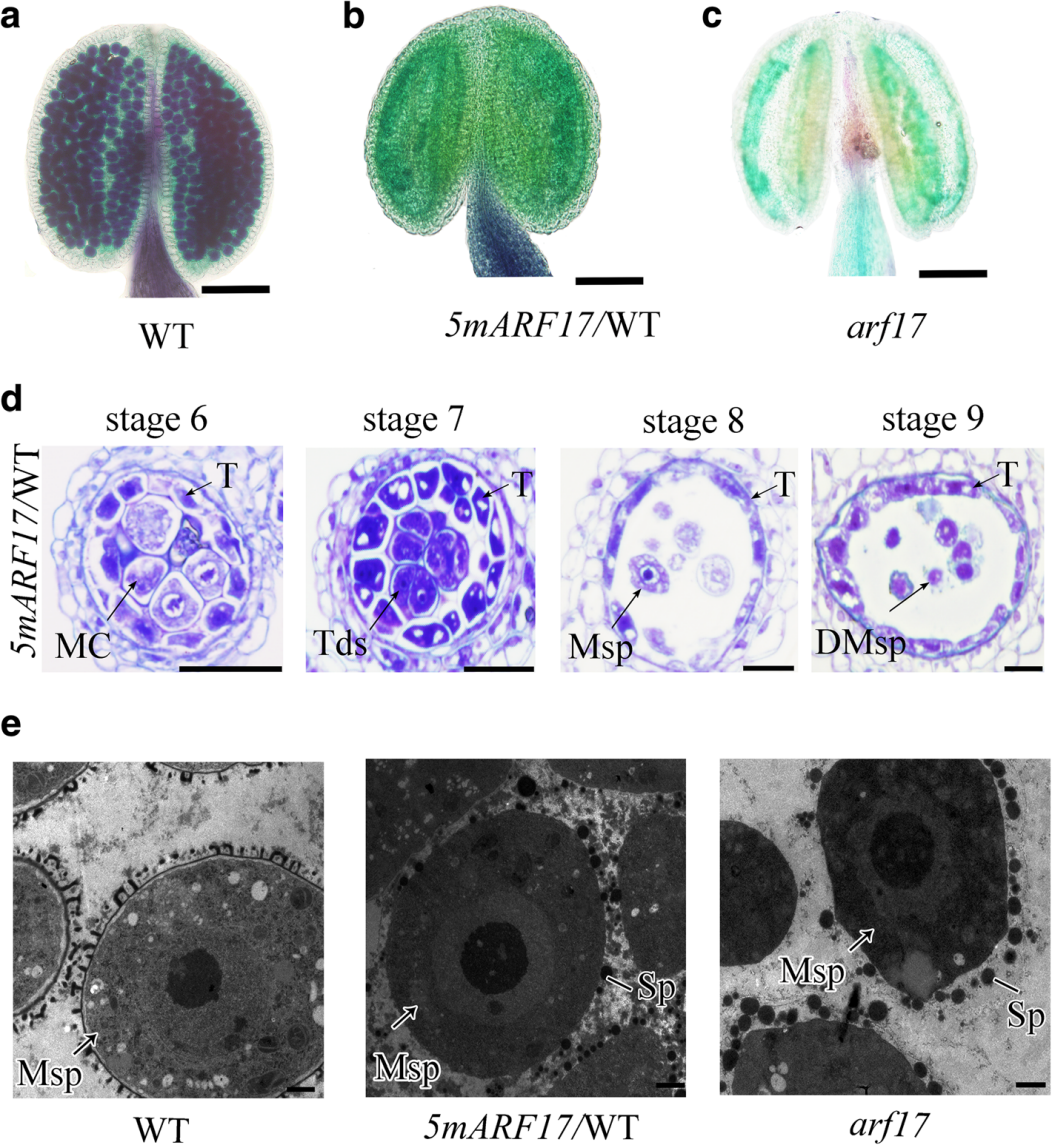
*PromoterARF17::5mARF17* plants show complete male sterility. **a**-**c** Alexander’s staining shows mature pollen in the anthers of a WT (**a**), a *promoterARF17::5mARF17* (*5mARF17/*WT) plant (**b**) and an *arf17* mutant (**c**). Bars = 50 μm. (**d**) Semi-thin sections of the anthers of a *5mARF17/*WT plant. Bar = 20 μm. (**e**) TEM showing the microspore structures of WT, *5mARF17* and *arf17* mutant plants at anther stage 8. Bar = 5 μm. DMsp, degenerated microspore; MC, meiotic cell; Msp, microspore; T, tapetum; Tds, tetrads

*ARF17* is among the *miR160* targets in *Arabidopsis*, and *promoterARF17::5mARF17* (*5mARF17*/WT) transgenic plants are sterile [20, 21]. To better understand the role of ARF17 in male reproduction, we performed the same *5mARF17* construction and obtained *5mARF17/*WT transgenic plants, which showed complete sterility as previously described (Additional file 1: Figure S1a-c; [20]). Alexander staining showed no mature pollen in the *5mARF17/*WT plants, which was similar to observations in *arf17* mutants (Fig. 2a-c). Reciprocal crosses with WT indicated that female fertility was not affected. Half of the F_1_ generation of *5mARF17/*WT plants pollinated with WT showed the male-sterile phenotype. PCR demonstrated that all these male-sterile plants contained the *5mARF17* fragment and that none of the fertile plants contained this fragment (Additional file 1: Figure S1d). These results suggest that *5mARF17* acts as a dominant gene for male sterility. Semi-thin sections showed no significant defects in anther development in the *5mARF17/*WT plants before stage 6. Tetrads formed normally in the *5mARF17/*WT plants at stage 7 (Fig. 2d), but the callose cell wall was much thinner than that in the WT plants (Additional file 1: Figure S1e-g). Tapetum development was also abnormal in the *5mARF17/*WT plants (Fig. 2d) but was less vacuolated than that in the *arf17* mutants (Fig. 1a). At stage 8, individual microspores were observed in the *5mARF17/*WT plants (Fig. 2d). Transmission electron microscopy (TEM) revealed that the exine of the *5mARF17/*WT plants was abnormal (Fig. 2e). After stage 8, the microspores became vacuolated and then degraded in the *5mARF17* plants (Fig. 2d). Therefore, both the tapetum and tetrad were defective in the *5mARF17/*WT plants, which led to pollen rupture and male sterility.

### *5mARF17* does not affect the expression of native *ARF17*

**Fig. 3:**
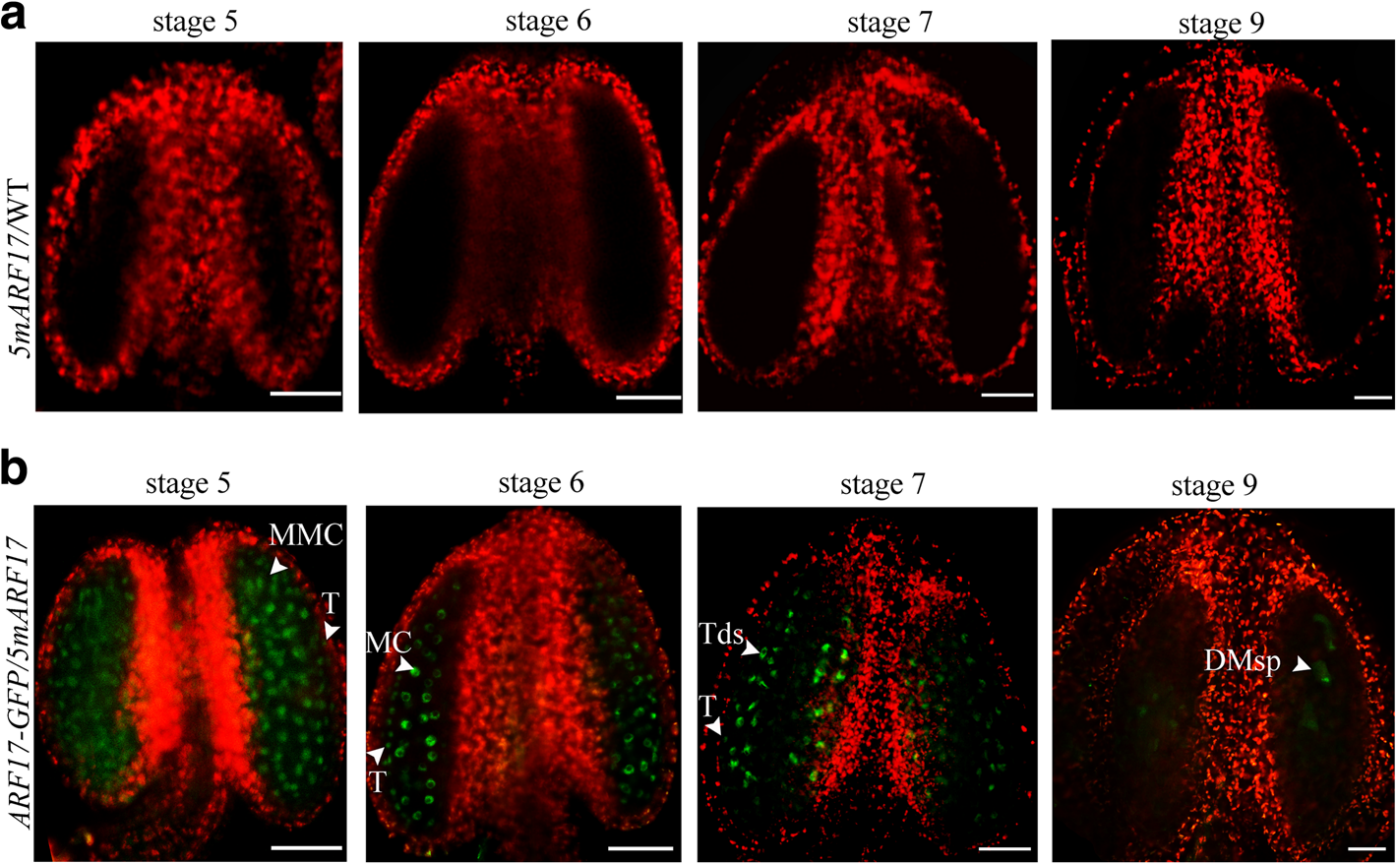
Expression of native ARF17 was not affected in *5mARF17*/WT plants. **a** and **b** Fluorescence confocal images indicating GFP signals in the anthers of a *5mARF17*/WT plant (**a**) and an *ARF17-GFP/5mARF17* plant (**b**). Green is the GFP signal (530 nm), and red is chlorophyll autofluorescence. Bars = 50 μm. DMsp, degenerated microspore; MC, meiotic cell; MMC, microspore mother cell; T, tapetum; Tds, tetrads

The phenotype of the *5mARF17/*WT plants regarding male development was similar to that of the *arf17* mutants, except the tapetal cells (Fig. 1, Additional file 1: Figure S1a-c, Fig. 2d and e). To determine whether the *5mARF17* transgene affected the expression of the native *ARF17*, *5mARF17/*WT plants were pollinated with pollen from *ARF17-GF*P/*arf17* plants to obtain *ARF17-GFP/5mARF17* plants. Because ARF17-GFP can complement the *arf17* phenotype, the ARF17-GFP signal represents the expression of native ARF17. In the *5mARF17*/WT plants, no GFP signals were observed (Fig. 3a). In the *ARF17-GFP/5mARF17* plants, the ARF17-GFP signals were detected in microsporocytes, tetrads and microspores (Fig. 3b). The expression pattern of ARF17-GFP in the *ARF17-GFP/5mARF17* plants was similar to that in the *ARF17-GFP/arf17* plants (Fig. 1c), which represented the native expression. Thus, the *5mARF17* transgene did not affect the expression of the native ARF17.

### *5mARF17* cannot rescue the sterility of *arf17*

**Fig. 4:**
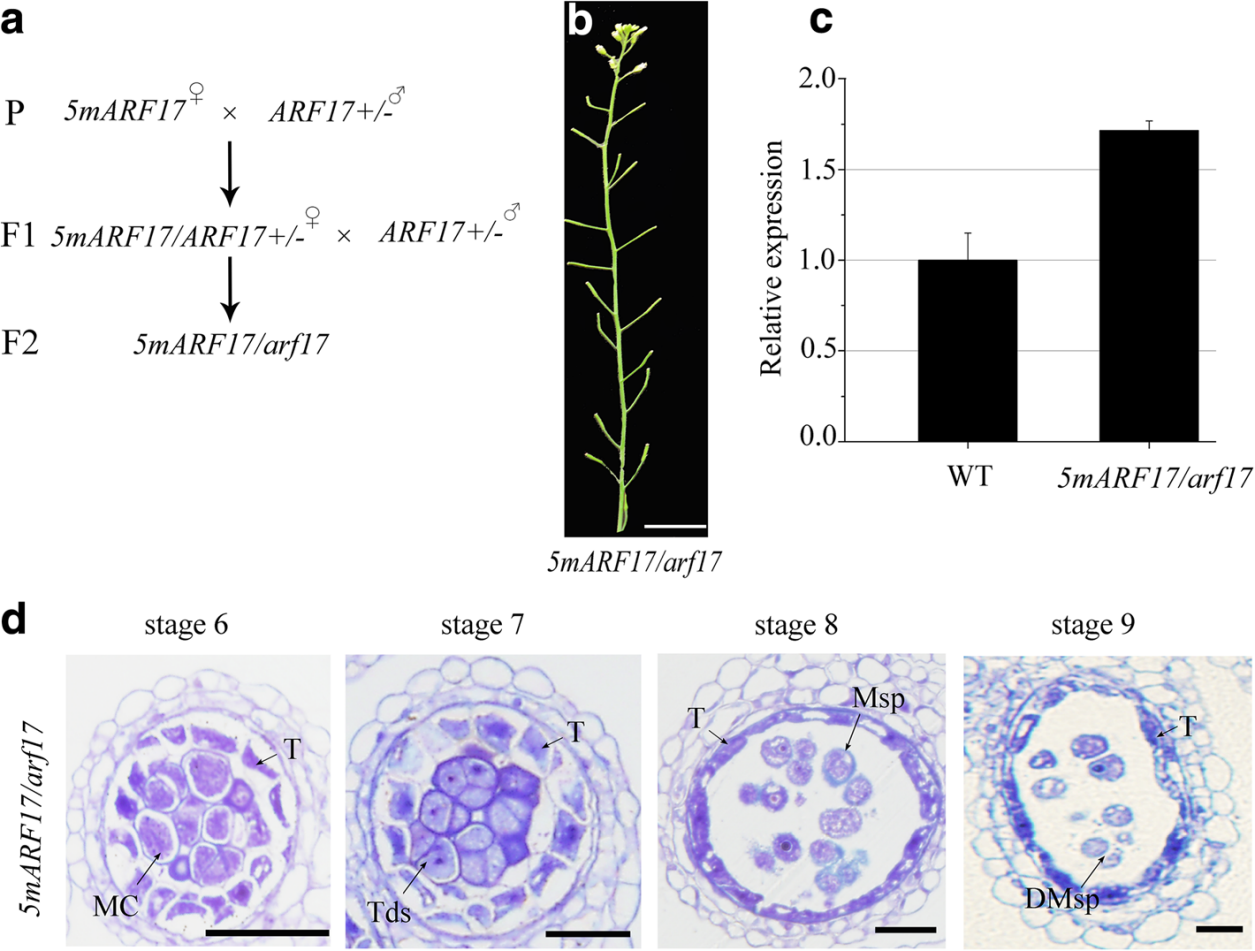
Phenotype of *5mARF17/arf17* mutants. **a** The schematic diagram indicates the steps to obtain a *5mARF17/arf17* plant. **b** A 35-day-old *5mARF17/arf17* plant. Bar = 2 cm. **c** Quantitative RT-PCR analysis of *ARF17* expression in WT and *5mARF17/arf17* buds*.* The level of *ARF17* was normalized to that tubulin and compared with that of WT. Error bars indicate SD and were calculated from three biological replicates. **d** Semi-thin sections of anthers in a *5mARF17/arf17* plant. Bar = 20 μm. DMsp, degenerated microspore; MC, meiotic cell; Msp, microspore; T, tapetum; Tds, tetrads

The *5mARF17* transgene encodes the same amino acid sequence as does *ARF17* [20]. To investigate whether *5mARF17* fulfilled the same function as *ARF17*, we crossed *5mARF17/*WT plants with pollen from a heterozygous *ARF17*/*arf17* plant. The F_1_ plants had the *5mARF17* transgene in the *ARF17*/*arf17* background and were further crossed with an *ARF17/arf17* plant to obtain a plant with the *5mARF17* transgene and an *arf17* background (*5mARF17/arf17)* (Fig. 4a, Additional file 2: Figure S2a and b). We found that the *5mARF17/arf17* plants remained male sterile (Fig. 4b). The results of qRT-PCR showed that the expression level of *ARF17* in the *5mARF17/arf17* plants was higher than that in the WT (Fig. 4c). Semi-thin sections of the anthers of the *5mARF17/arf17* plants showed similar defects in the tapetum and microspores to those in the *5mARF17/*WT plants (Figs. 2d and 4d). However, the tapetum showed less vacuolation than did the *arf17* mutant (Fig. 1a)*.* Therefore, the *5mARF17* transgene could partly rescue the tapetum development rather than recover male fertility in the *arf17* mutants. In our previous work, the same promoter of *ARF17* used to construct *pARF17::ARF17* complemented the *arf17* mutant phenotype [17]. These results indicate that the escape of *ARF17* expression from *miR160* regulation leads to developmental defects in the anthers as well as male sterility.

### ARF17 protein over-accumulates in the tapetum and microsporocytes of *5mARF17*/WT plants

**Fig. 5:**
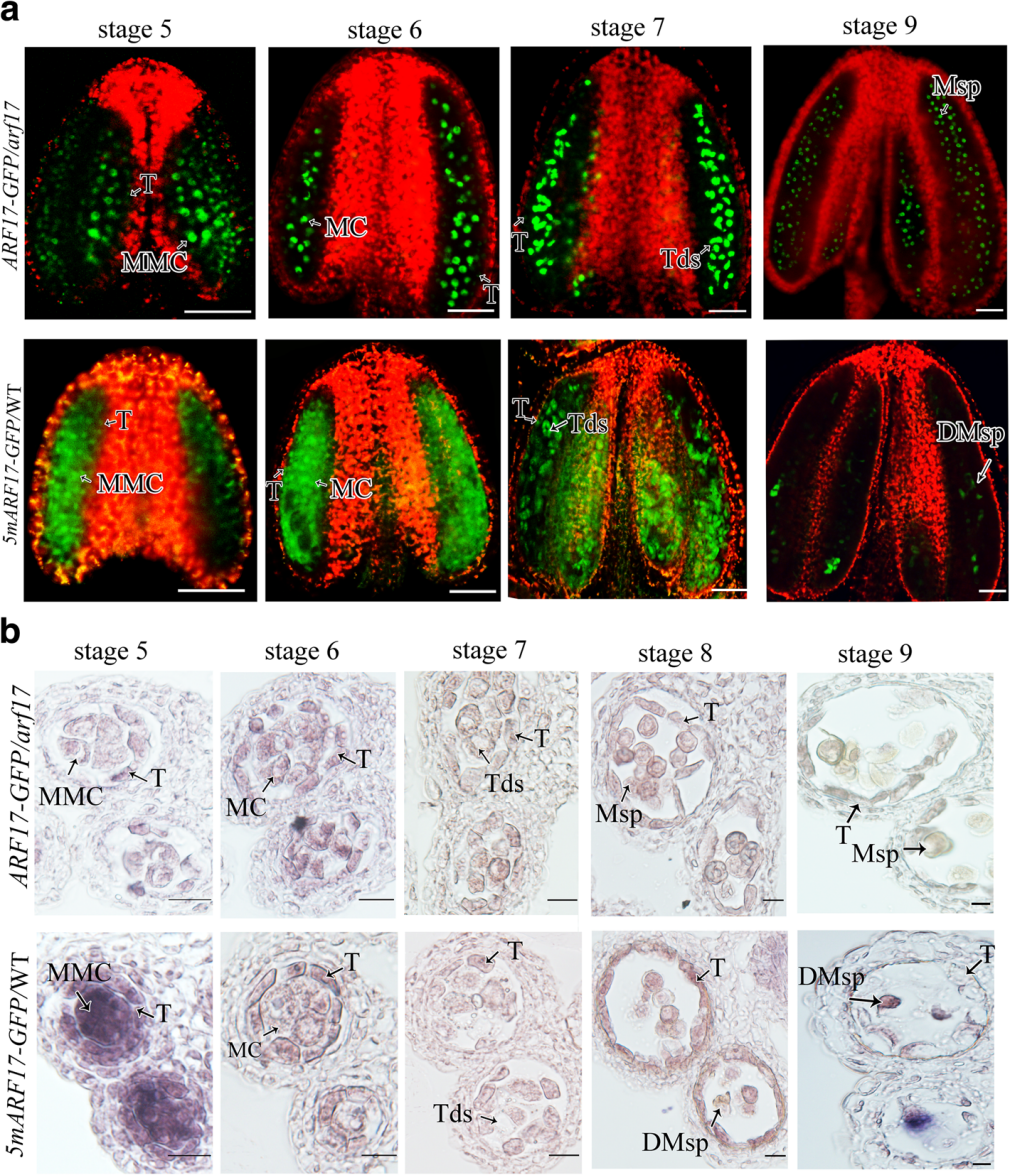
GFP signals in *ARF17-GFP/arf17* and *5mARF17-GFP/*WT anthers. **a** Fluorescence confocal images indicating GFP signals in the anthers of *ARF17-GFP*/*arf17* and *5mARF17-GFP/*WT plants. Bar = 50 μm. Green is the GFP signal (530 nm), and red is chlorophyll autofluorescence. **b** Immunohistochemical assays of GFP in the anthers at stages 5–10 of *ARF17-GFP/arf17* and *5mARF17-GFP/*WT plants. Bar = 20 μm. DMsp, degenerated microspore; MC, meiotic cell; MMC, microspore mother cell; Msp, microspore; T, tapetum; Tds, tetrads

In a previous study, *miR160* failed to cleave *5mARF17* mRNA, and *ARF17/5mARF17* mRNA over-accumulated in *5mARF17*/WT plants [20]. To determine whether ARF17 protein also over-accumulated in the *5mARF17*/WT plants, we constructed a *promoterARF17::5mARF17-GFP* construct and then introduced it into a WT plant to obtain *5mARF17-GFP/*WT transgenic plants. The *5mARF17-GFP/*WT plants also showed a male-sterile phenotype and a similar segregation ratio as that of the *5mARF17/*WT plants (Additional file 3: Figure S3a-c). Confocal laser scanning microscopy was used to investigate GFP signals. During anther stages 5 to 7, 5mARF17-GFP protein was expressed in the microsporocytes, microspores and tapetum. However, the GFP signals in the *5mARF17-GFP/*WT plants were much more diffuse than those of the ARF17-GFP plants (Fig. 5a). Defects in the tapetum and microsporocytes could lead to the diffuse pattern of the ARF17-GFP signal in the *5mARF17-GFP/*WT plants. To confirm whether ARF17 over-accumulated in the *5mARF17/*WT plants, an immunohistochemical assay was employed using a GFP antibody to detect the accumulation of both 5mARF17-GFP in the *5mARF17/*WT plants and ARF17-GFP in the *ARF17-GFP/arf17* plants. In the *ARF17-GFP/arf17* plants, the ARF17-GFP signal was observed in microsporocytes, tapetum, tetrads and microspores from stage 5 to stage 9 (Fig. 5b). In the *5mARF17-GFP*/WT plants, 5mARF17-GFP proteins were more highly accumulated in the tapetum and microsporocytes than were the ARF17-GFP proteins in the *ARF17-GFP/arf17* plants at stage 5. At stages 6–8, 5mARF17-GFP proteins continued to be expressed in the tapetum and microspores. Then, we harvested young buds (anther stage 5) from both *5mAFR17*/WT and WT plants and investigated the expression of *ARF17* via qRT-PCR. The results of the qRT-PCR show that the expression of *ARF17* in the *5mARF17*/WT plants is higher than that in the WT plants (Additional file 4: Figure S4b). These results indicated that 5mARF17 protein over-accumulated in both the tapetum and microsporocytes at anther stage 5 in the *5mARF17/*WT plants. This finding suggests *miR160* is important for normal anther development in regulating ARF17 expression at an early anther stage.

### Transgenic plants containing *5mARF17* driven by the tapetum-specific promoter *A9* are male sterile

**Fig. 6:**
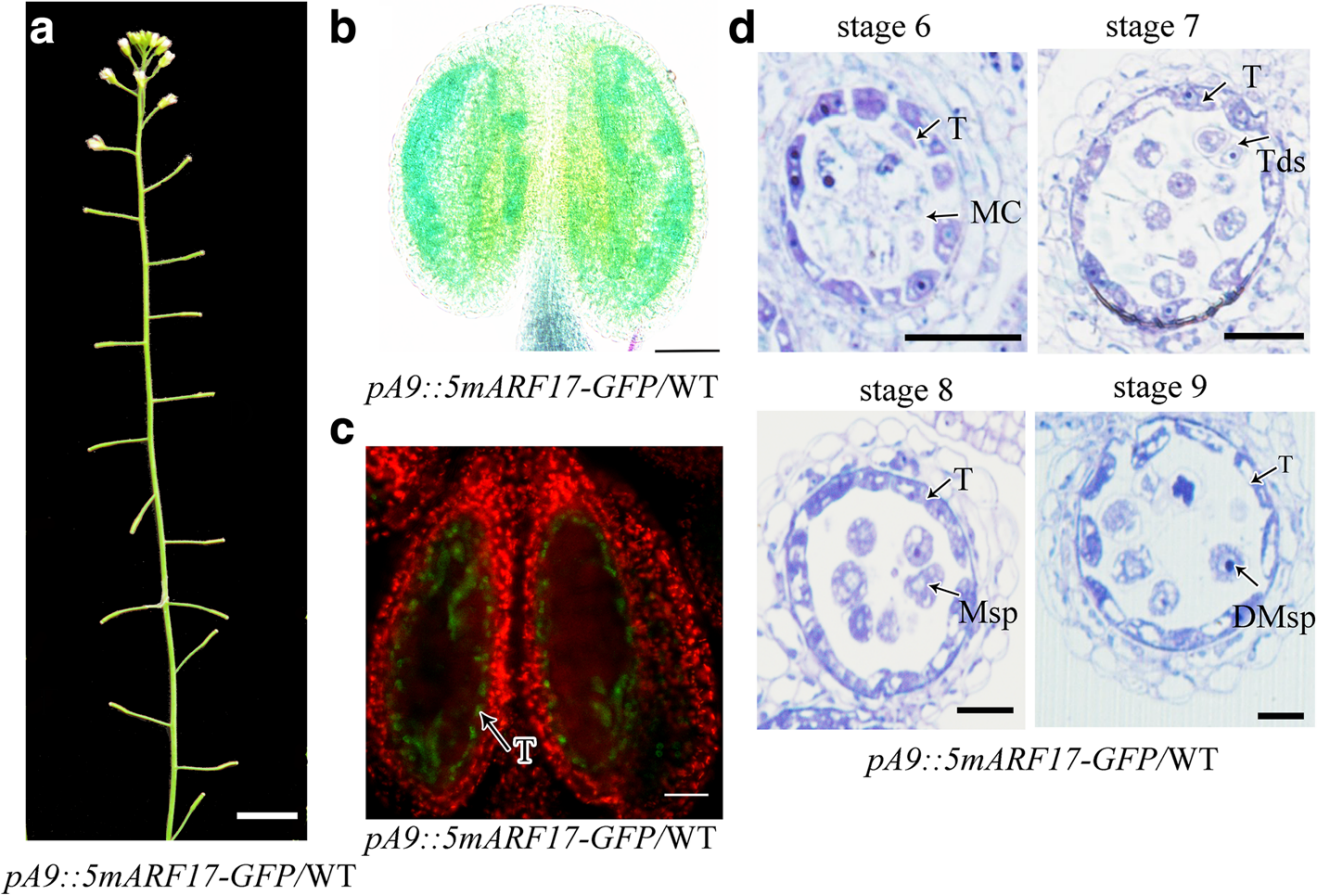
Phenotype of a *promoterA9::5mARF17-GFP/*WT plant. **a** A 35-day-old *promoterA9::5mARF17-GFP* (*pA9::5mARF17-GFP/*WT) plant. Bar = 2 cm. **b** Alexander’s staining of the anthers of a *pA9::5mARF17-GFP*/WT plant*.* Bar = 200 μm. **c** GFP signals were observed exclusively in the tapetal cells of *pA9::5mARF17-GFP*/WT plants. Bar = 50 μm. **d** Semi-thin sections of the anthers of a *pA9::5mARF17-GFP*/WT plant showing pollen development from anther stages 6–9. Bar = 20 μm. DMsp, degenerated microspore; MC, meiotic cell; Msp, microspore; T, tapetum; Tds, tetrads

Both the tapetum and microsporocytes contribute to pollen formation. To determine whether the overexpression of ARF17 in the tapetum leads to microspore abortion and male sterility, we constructed a *promoterA9::5mARF17-GFP* construct and introduced it into a WT plant. The *A9* promoter can drive gene expression specifically in the tapetum from anther stages 5 to 9 [22, 23]. We found that the *promoterA9::5mARF17-GFP/*WT transgenic plants (*pA9::5mARF17-GFP*/WT) were male sterile (Fig. 6a, b), and the segregation ratio was similar to that of *5mARF17/*WT plants (Additional file 4: Figure S4a). The results of qRT-PCR showed that the expression of *ARF17* in the *pA9::5mARF17-GFP/*WT plants was higher than that in the WT plants (Additional file 4: Figure S4c). The GFP signal in the *pA9::5mARF17-GFP/*WT plants was restricted to the tapetum (Fig. 6c). Semi-thin sections showed the defects in tapetum development and pollen formation of the *pA9::5mARF17-GFP/*WT plants; these defects were similar to those of *5mARF17/*WT plants (Figs. 2d and 6d). These results show that the over-accumulation of ARF17 in the tapetum was sufficient to lead to tapetum defects and plant sterility.

### Identification and overexpression of up-regulated genes in *5mARF17*/WT plants

Our previous work demonstrated that ARF17 functions as a transcriptional activator [17]. To identify the affected genes in *5mARF17/*WT plants, we performed a microarray analysis using young buds from the *5mARF17/*WT and WT plants. A total of 755 genes were up-regulated in *5mARF17* plants compared with WT plants (Additional file 5: Table S3). Based on the BAR information (http://bar.utoronto.ca), 220 (29.14%) genes are ectopically expressed, and 339 (44.90%) are overexpressed in *5mARF17*/WT plants (anther stage 5–7) (Additional file 5: Table S3). PANTHER (Protein Analysis Through Evolutionary Relationships, http://pantherdb.org) [24] analysis indicated that 316 known genes are overexpressed in *5mARF17/*WT buds (Fig. 6a; Additional file 6: Table S4), with 77 of the 316 genes associated with nucleic acid binding (24.4%). In this group, DNA helicase, RNA binding protein, ribosomal protein and histones were highly enriched (Additional file 6: Table S4). These results indicate that overexpression of ARF17 may lead to alterations of normal gene expression, resulting in disruption of anther and tapetum development.

**Fig. 7:**
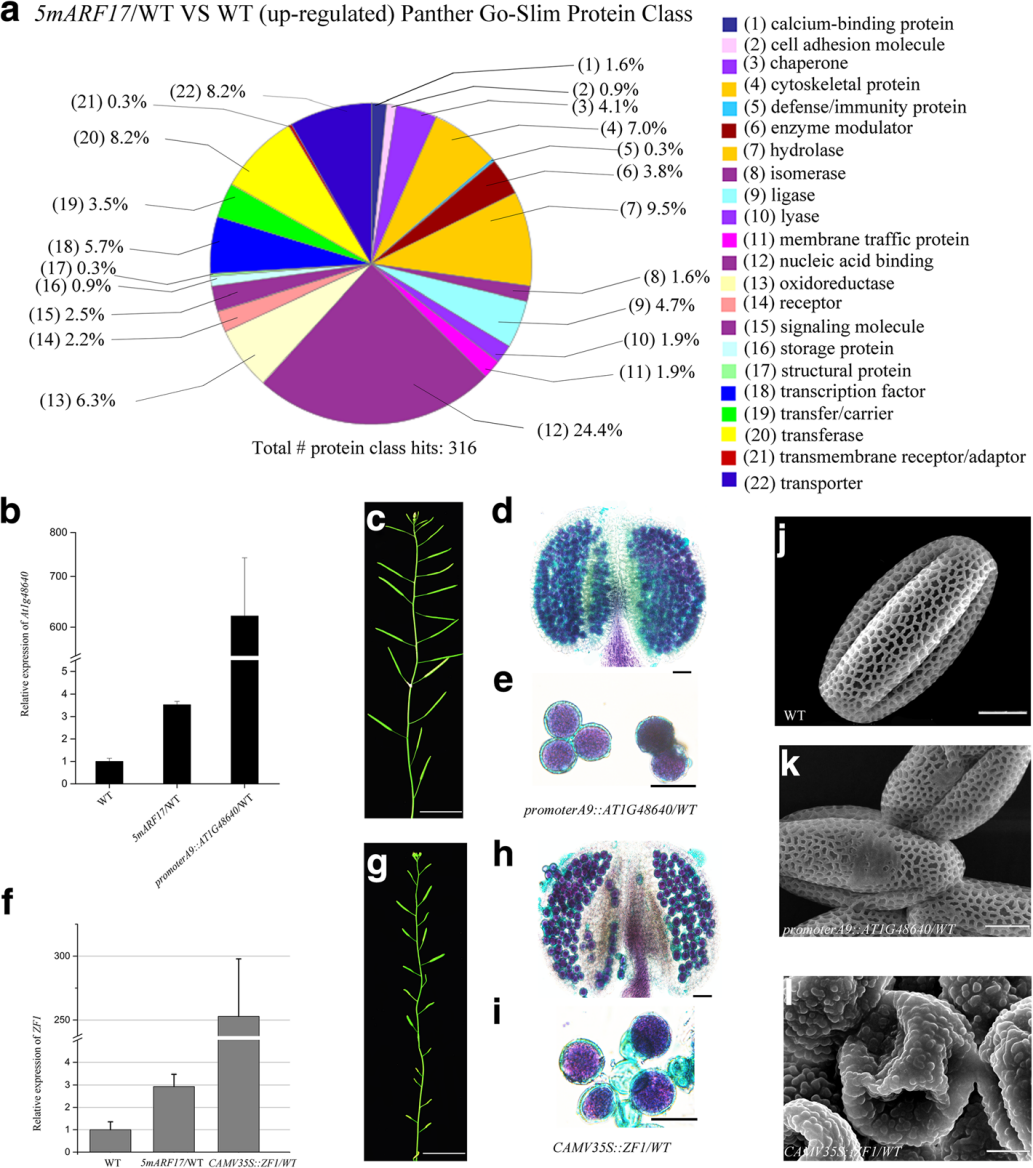
Up-regulated genes in a *5mARF17*/WT plant. **a** Panther Go-Slim protein classes of up-regulated genes in *5mARF17*/WT plants compared with those in WT. The total hits included 316 genes. The percentage of each term is the clustered gene number of the total protein class hits: calcium binding (5 genes), cell adhesion molecule (3 genes), chaperone (13 genes), cytoskeletal protein (22 genes), defense immunity protein (1 gene), enzyme modulator (12 genes), hydrolase (30 genes), isomerase (5 genes), ligase (15 genes), lyase (6 genes), membrane traffic protein (6 genes), nucleic acid binding (77 genes), oxidoreductase (20 genes), receptor (7 genes), signaling molecule (8 genes), storage protein (3 genes), structural protein (1 gene), transcription factor (18 genes), transfer/carrier protein (11 genes), transferase (26 genes), transmembrane receptor regulatory/adaptor protein (1 gene), and transporter (26 genes). **b**-**l** The phenotypes of *promoterA9::AT1G48640* and *CAMV35s::ZF1* plants. **b** Quantitative RT-PCR analysis of *AT1G48640* expression in WT, *5mARF17*/WT and *promoterA9::AT1G48640/*WT buds. **c** A 35-day-old *promoterA9::AT1G48640/*WT plant. Bar = 2 cm. **d** and **e** Alexander’s staining of the anthers and pollen grains of a *promoterA9::AT1G48640/*WT plant. Bars = 20 μm. **f** Quantitative RT-PCR analysis of *ZF1* expression in WT, *5mARF17*/WT and *CAMV35s::ZF1/*WT buds. **g** A 35-day-old *CAMV35s::ZF1/*WT plant. Bar = 2 cm. **h** and **i** Alexander’s staining of the anthers and pollen grains of a *CAMV35s::ZF1/*WT plant. Bars = 20 μm. **j**-**l** Scanning electron microscopy (SEM) showing the pollen wall structures of a WT plant (**j**), a *promoterA9::AT1G48640/*WT plant (**k**) and a *CAMV35s::ZF1/*WT plant (**l**). Bars = 5 μm. The levels of *AT1G48640* and *ZF1* were normalized to those of tubulin and compared with those of WT. Error bars indicate SD and were calculated from three biological replicates

To test whether the expression of some of the up-regulated genes in the *5mARF17*/WT plants affect anther development and pollen formation, we fused some of the genes to *CaMV35S* or *A9* promoters, and the resulting constructs were introduced into WT plants. *AT1G48640* encodes a transmembrane amino acid transporter family gene that is involved in root development [25]. 12/12 transgenic lines with over-expression of *AT1G48640* were fertile in *promoterA9::AT1G48640/*WT transgenic plants (Additional file 7: Figure S6B, Fig. 7b and c), but their pollen grains adhered together (Fig. 7d, e), and scanning electron microscopy (SEM) showed defects in pollen wall structure in those plants (Fig. 7j, k). Zinc-finger protein 1 (ZF1) functions as a transcriptional repressor and is involved in the inhibition of plant growth under abiotic stress conditions [25–27]. 8/8 transgenic lines with over-expression of *ZF1* showed reduced fertility in *CaMV35s::ZF1/*WT plants (Additional file 7: Figure S6b, Fig. 7f and g). Some pollen was aborted and adhered together in the *CaMV35s::ZF1/*WT plants (Fig. 7h, i), and SEM showed that pollen wall formation was also defective in these plants (Fig. 7j, l).

## Discussion

### ARF17 is important for both tapetum and microsporocyte development

ARF17 is a member of the ARF family in *Arabidopsis*. In *arf17* mutants, all pollen is ruptured, and plants show male sterility [17]. During anther development, the microsporocyte/tetrad determines the pollen wall pattern, whereas tapetal cells provide both nutrition and pollen wall materials for pollen formation. ARF17 is expressed in microsporocytes and tetrads during anther development and directly regulates the expression of *CALS5* for both tetrad wall synthesis and exine pattern formation [17]. In this work, ARF17 expression was also detected in the tapetum based on the ARF17-GFP signal and immunocytochemical assay results (Figs. 1c and 5b). The ARF17-GFP signals were detected in the tapetum at an early stage for a short period. In addition, the signal was much weaker than that in the microsporocytes/tetrads (Fig. 1c), with the weaker signal the primary reason that the expression was not detected in the tapetum in previous studies [17]. However, the short expression period was important for the development of the tapetum, with the tapetum clearly defective in the *arf17* mutants (Fig. 1a). After stage 4, a key genetic pathway of DYT1-TDF1-AMS is important for tapetum development and pollen wall formation [28–31]. DYT1 is the earliest transcription factor required for tapetum development and is initially detected at approximately stage 5 [28]. In the *arf17* mutants, *DYT1*, *TDF1*, and *AMS* expression was not significantly affected (Additional file 8: Figure S5a), and in *dyt1* and *tdf1* mutants, *ARF17* transcription was not dramatically affected (Additional file 8: Figure S5b). Therefore, ARF17 plays a critical role in early tapetum development and may be independent of the DYT1-TDF1-AMS pathway.

### Overexpression of ARF17 in *5mARF17*/WT plants leads to defective microsporocytes and tapetum

Previous studies have shown that *5mARF17/*WT plants are sterile [20]. Reciprocal crosses with wild-type plants showed that the female parts are not affected in the *5mARF17/*WT plants (Additional file 1: Figure S1d). The *5mARF17/*WT plants had no mature pollen inside anther locules, and both the tapetum and microsporocytes/tetrads were defective (Fig. 2a-e). Overall, the cellular defects of anther development were similar between the *arf17* and *5mARF17/*WT plants. However, the defective tapetum development in the *arf17* mutants is more severe than that in the *5mARF17*/WT plants after stage 6 (Additional file 7: Figure S6a). In the *arf17* mutants, the loss of ARF17 function led to pollen rupture and male sterility. In *5mARF17/*WT plants, the transgene *5mARF17* did not affect the expression of the native ARF17 (Fig. 3a and b). Although *5mARF17* encodes the same protein as does *ARF17*, it could not complement the male sterility of *arf17* mutants (Fig. 4b). Hence, the mechanisms that lead to the male sterility of both *5mARF17/*WT and *arf17* plants are different. In the *5mARF17*/WT plants, ARF17 protein over-accumulated in the tapetum and microsporocytes (Fig. 5a, b). The tapetum-specific over-accumulation of ARF17 was sufficient to cause male sterility, which was similar to that observed for the *5mARF17*/WT plants (Fig. 6a-d). Therefore, the overexpression of ARF17 in the tapetum of the *5mARF17*/WT plants prevents normal tapetum function. The microsporocytes/tetrads are also defective in the *5mARF17/*WT plants. It is likely that the overexpression of ARF17 in the microsporocytes/tetrads of the *5mARF17*/WT plants leads to its male sterility. ARF17 directly regulates the expression of *CALS5*, which is needed for tetrad wall formation. In the *arf17* mutants, the expression of *CALS5* is significantly reduced [17]. The *5mARF17/*WT plants also show defective tetrad walls and reduced expression of *CALS5* (Additional file 1: Figure S1e-h). It is likely that these phenomena are a side effect of defective microsporocyte/tetrad development in the *5mARF17/*WT plants. The results of the microarray analysis showed that *5mARF17* activates the ectopic expression of many genes in the anthers of the *5mARF17/*WT plants. Overexpression of two of these genes, *AT1G48640* and *ZF1*, slightly affected pollen formation and plant fertility (Fig. 7b-l). Therefore, the male sterility of the *5mARF17/*WT plants resulted from the ectopic expression and overexpression of these genes, including *AT1G48640* and *ZF1*.

### Expression of ARF17 is precisely regulated during anther development

ARF17 is among the targets of *miR160* [20, 32], and the knockout of *ARF17* leads to male sterility [17]. In this work, we demonstrated that without *miR160* control in *5mARF17*/WT plants, ARF17 was overexpressed, which led to male sterility. Therefore, the expression level of ARF17 is critical for pollen development. The expression pattern of 5mARF17-GFP was apparently similar to that of ARF17-GFP (Fig. 5b). Therefore, the *ARF17* promoter determined the cell and tissue specificity of ARF17 expression, whereas *miR160* controlled the level of ARF17 expression. In *Arabidopsis*, three precursors, including *miR160a*, *miR160b* and *miR160c*, can produce *miR160* [20]. In humans, the TRISTETRAPROLINE (TTP) protein is a member of the RNA-induced silencing complex (RISC) [33, 34]. AtTTP is an ortholog of hTTP in *Arabidopsis* and is expressed in microsporocytes, tetrads and tapetal cells, and *AtTTP* expression highly overlapped with that of *ARF17*. Overexpression of *AtTTP* decreases the level of mature *miR160*, whereas the expression of *ARF17* increases it [35]. Thus, AtTTP is involved in *miR160* maturation for the fine regulation of ARF17 for pollen formation during anther development.

## Conclusion

ARF17 is the target of *miR160*, and *5mARF17/*WT plants show male sterility as do *arf17* plants. In this study, we showed that ARF17 plays an essential role in anther development and pollen formation. Without *miR160* regulation, the expression pattern of *ARF17* in the anthers is not affected, but its expression level is significantly elevated in *5mARF17*/WT plants. The overexpression of ARF17 in *5mARF17* plants leads to defects in pollen formation and plant sterility.

## Methods

### Plant materials and growth conditions

In this study, *Arabidopsis* (*Arabidopsis thaliana*) wild-type (WT), transgenic and mutant plants in the Col-0 ecotype background were used. *arf17* mutants and *5mARF17*/WT plants were preserved in the laboratory of Z.N. Yang. Seeds were sown on vermiculite and allowed to imbibe for 2 to 3 days. Plants were grown under conditions of 16 h light/8 h darkness in a growth chamber for approximately 22 days.

### Microscopy

The plants were imaged with a Cyber Shot T-20 digital camera (Sony). Alexander staining solution was prepared as described [23]. The anthers were stained for approximately 2–12 h at room temperature (RT, 22 °C). For semi-thin sections, the flower buds were fixed and embedded in Spurr’s resin. Semi-thin sections were prepared by cutting the buds to a thickness of 1 μm followed by incubation in a 0.01% toluidine blue/sodium borate solution for 5–10 min at 42 °C, after which the sections were washed with water. The sections were observed with an Olympus BX51 microscope under bright-field microscopy.

### Fluorescence microscopy

For callose staining, anthers at the tetrad stage were squeezed onto a slide and stained with toluidine blue as described previously [17]. GFP signals in the anthers of *ARF17-GFP/arf17*, *5mARF17-GFP/*WT, and *ARF17-GFP/5mARF17* plants were observed using a Carl Zeiss confocal laser scanning microscope (LSM 5 PASCAL).

### Electron microscopy

For TEM examination, flower buds were fixed in 0.1 M phosphate buffer (pH 7.2) with 2.5% glutaraldehyde (*v*/v) and then washed several times with PBS (pH 7.4), followed by dehydration with ethyl alcohol and replacement by propylene epoxide. The samples were embedded in Spurr’s resin and polymerized for 48 h at 60 °C as described previously [23, 30]. Then, TEM microscopy (JEOL, Japan) was used to observe the slides. For SEM observations, fresh pollen grains were coated with 8 nm of gold and observed under a JSM-840 microscope (JEOL) [23].

### Plasmid construction and identification of transgenic plants

The *promoterARF17::5mARF17* genomic region was cloned using *ARF17* primers (ProARF17-F/R) and (5mARF17g-F/R) to complement the wild type (WT) in accordance with Mallory identification [20]. The *promoterA9::5mARF17-GFP* fragment was constructed with the *A9* promoter (ProA9-F/R) and the *5mARF17* genomic region to complement the WT. For GFP fusion, the *5mARF17* genomic fragment without a stop codon was cloned into a modified GFP-pCAMBIA1300 vector using primers (5mARF17g-F/5mARF17g-GFP-R) to complement the WT. The *promoterA9::AT1G48640* fragment was constructed with the *A9* promoter (ProA9-F/R) and the *AT1G48640* CDS (4864-F/4864-R) region transferred into the WT. The *CaMV35:ZF1* fragment was constructed with the *CAMV35* promoter (35 s–F/35 s–R) and the *ZF1* CDS (ZF1-F/ZF1-R) region introduced into the WT. These fragments were amplified using KOD polymerase (Takara Biotechnology) and cloned into the pCAMBIA1300 and GFP-pCAMBIA1300 vectors (CAMBIA). The fragments were verified by sequencing. The plasmids were transformed into *Agrobacterium tumefaciens* (GV3101) and screened using 50 mg/ml kanamycin, 40 mg/ml gentamicin and 50 mg/ml rifampicin. The bacteria containing the plasmid constructs were introduced into the flower buds. The transgenic plants were selected using 20 mg/l hygromycin. The primer sequences are listed in Additional file 9: Table S1.

### Immunohistochemical staining

For immunohistochemical staining, flower buds were fixed in formaldehyde/acetic acid for 1 day, dehydrated in an ethanol gradient and embedded in wax. Sections that were 8 mm thick were prepared using a rotary microtome (MR2; RMC). The sections were incubated in boiled citrate buffer (pH 6.4) for 10–15 min after rehydration and then cooled to RT. The slides were washed twice in PBS (pH 7.4) for 5 min. Then, the sections were blocked with 5% BSA in PBS for 30 min to 1 h. Rabbit anti-6 × GFP antibodies (Thermo Scientific) were diluted 1:100 in PBS (pH 7.4), and the slides were then incubated at 4 °C overnight. Then, the slides were washed three times in Tris-buffered saline solution (TBS; pH 7.4) for 5 min and incubated with anti-rabbit-AP antibodies diluted 1:200 in TBS (pH 7.4) for 1 h at RT. The slides were washed three times in TBS for 5 min, and BCIP/NBT solution (CWBIO) was used for colorimetric detection at RT.

### Quantitative RT-PCR

Total RNA was extracted from the flower buds of the WT, transgenic and mutant plants using a TRIzol kit (Invitrogen). In accordance with the manufacturer’s instructions (Toyobo), first-strand complementary DNA (cDNA) was synthesized. Quantitative RT-PCR was performed using an ABI PRISM 7300 detection system (Applied Biosystems) with SYBR Green I master mix (Toyobo). The primers ARF17qRT-F/R were used to detect the expression level of *ARF17*. The relative expression levels were calculated according to the cycle numbers. The qRT-PCR results are shown as the relative expression levels normalized to those of tubulin. The positive control was the tubulin gene (TUB-F/R), and three replicates were performed for each experiment. The relevant primer sequences are listed in Additional file 10: Table S2.

### Microarrays

Microarrays were performed according to a previously described procedure [30]. Young buds collected from WT and *5mARF17/*WT plants were immediately frozen in liquid nitrogen. A Low-RNA-Input Linear Amplification Kit (Agilent Technologies) was used to amplify and label the total RNA. 5-(3-Aminoallyl)-UTP (Ambion), Cy3 NHS ester (GE Healthcare Biosciences) and Cy5 NHS ester (GE Healthcare Biosciences) were applied following the manufacturers’ instructions. The labeled cRNA was purified using an RNeasy Mini Kit (Qiagen). According to the manufacturer’s instructions, each 44-K *Arabidopsis* oligo microarray slide was hybridized with 825 ng of Cy3-labeled cRNA and 825 ng of Cy5-labeled cRNA using a gene expression hybridization kit (Agilent) in a hybridization oven (Agilent). The slides were scanned using an Agilent Microarray Scanner (Agilent) and Feature Extraction software 10.7 (Agilent) with the default settings. Three biological replicates of independently grown materials were used. The raw data were normalized with a locally weighted scatter plot smoothing (Lowess) algorithm using Gene Spring Software 11.0 (Agilent).

## Additional files

Additional file 1: Figure S1. The phenotype, segregation analysis and expression of *CALS5* in *5mARF17/*WT plants. A-C A 35-day-old wild-type plant (WT) (A); *5mARF17*/WT plant (B) and *arf17* mutant (C). Bars = 2 cm. (D) Transmission efficiency of *5mARF17/*WT mutants*.* TE_f_: female transmission efficiency; TE_m_: male transmission efficiency; NA: no application. E-G Aniline blue staining of the callose from a WT plant (E); a *5mARF17*/WT plant (F); and an *arf17* mutant (G). Bars = 20 μm. (H) Quantitative RT-PCR analysis of *CALS5* expression in WT, *5mARF17* and *arf17* buds*.* The level of *CALS5* was normalized to that of tubulin and compared with that of WT. Error bars indicate SD and were calculated from three biological replicates. (TIFF 5996 kb)

Additional file 2: Figure S2. Identification of sequences in a *5mARF17/arf17* plant. A Genomic PCR analysis of a *5mARF17/arf17* plant background. B Clone sequencing of 5 bases in *5mARF17/arf17* plants. (TIFF 4304 kb)

Additional file 3: Figure S3. Phenotype and segregation analyses of *5mARF17-GFP/*WT plants. A and B A 35-day-old *5mARF17-GFP/*WT plant (A) and *ARF17-GFP/5mARF17* plant (B). Bars = 2 cm. (C) Transmission efficiency of a *5mARF17-GFP/*WT plant*.* TE_f_: female transmission efficiency; TE_m_: male transmission efficiency; NA: no application. (TIFF 1132 kb)

Additional file 4: Figure S4. Transmission efficiency of a *pA9::5mARF17-GFP/WT* plant. A Transmission efficiency of a *promoterA9::5mARF17-GFP/*WT (*pA9::5mARF17-GFP/*WT) plant*.* B Quantitative RT-PCR analysis of *ARF17* expression in WT and *5mARF17*/WT buds at stage 5. C Quantitative RT-PCR analysis of *ARF17* expression in WT and *pA9::5mARF17-GFP/*WT buds*.* The level of *ARF17* was normalized to that of tubulin and compared with that of WT. Error bars indicate SD and were calculated from three biological replicates. TE_f_: female transmission efficiency; TE_m_: male transmission efficiency; NA: no application. (TIFF 491 kb)

Additional file 5: Table S3. List of up-regulated genes in *5mARF17*/WT that are typically expressed or absent in WT buds. (XLSX 65 kb)

Additional file 6: Table S4. Protein classification of up-regulated genes by PANTHER. (XLS 116 kb)

Additional file 7: Figure S6. Semi-thin sections of anthers and PCR analysis. A Semi-thin sections of anthers in WT, *arf17* and *5mARF17*/WT plants from stage 5 to 9. Bar = 20 μm. B Identification of transgenic sequences in WT, *promoterA9:: AT1G48640*/WT and *CAMV35S::ZF1*/WT. DMsp, degenerated microspore; MC, meiotic cell; MMC, microspore mother cell; Msp, microspore; T, tapetum; Tds, tetrads. (TIFF 23380 kb)

Additional file 8: Figure S5. Relative expression of tapetum- and pollen-formation genes in *5mARF17*/WT and *arf17* plants. A Quantitative RT-PCR analysis of the expression levels of genes involved in tapetum and pollen development. B Quantitative RT-PCR analysis of *ARF17* expression in *dyt1* and *tdf1* mutants. The levels of *DYT1*, *TDF1*, *TEK*, *CDKG1*, *RPG1*, *MS188*, *MS1*, *ACOS5*, and *ARF17* were normalized to those of tubulin and compared with those of WT. Error bars indicate SD and were calculated from three biological replicates. (TIFF 937 kb)

Additional file 9: Table S1. List of primers used in clones and identification. (XLS 21 kb)

Additional file 10: Table S2 List of primers used in qRT-PCR. (XLS 22 kb)
