# Supplementary figures and images for: Fine regulation of ARF17 for anther development and pollen formation
Source: BMC Plant Biol. 2017 Dec 19;17:243. doi: 10.1186/s12870-017-1185-1 (PMC5735505; doi:10.1186/s12870-017-1185-1)

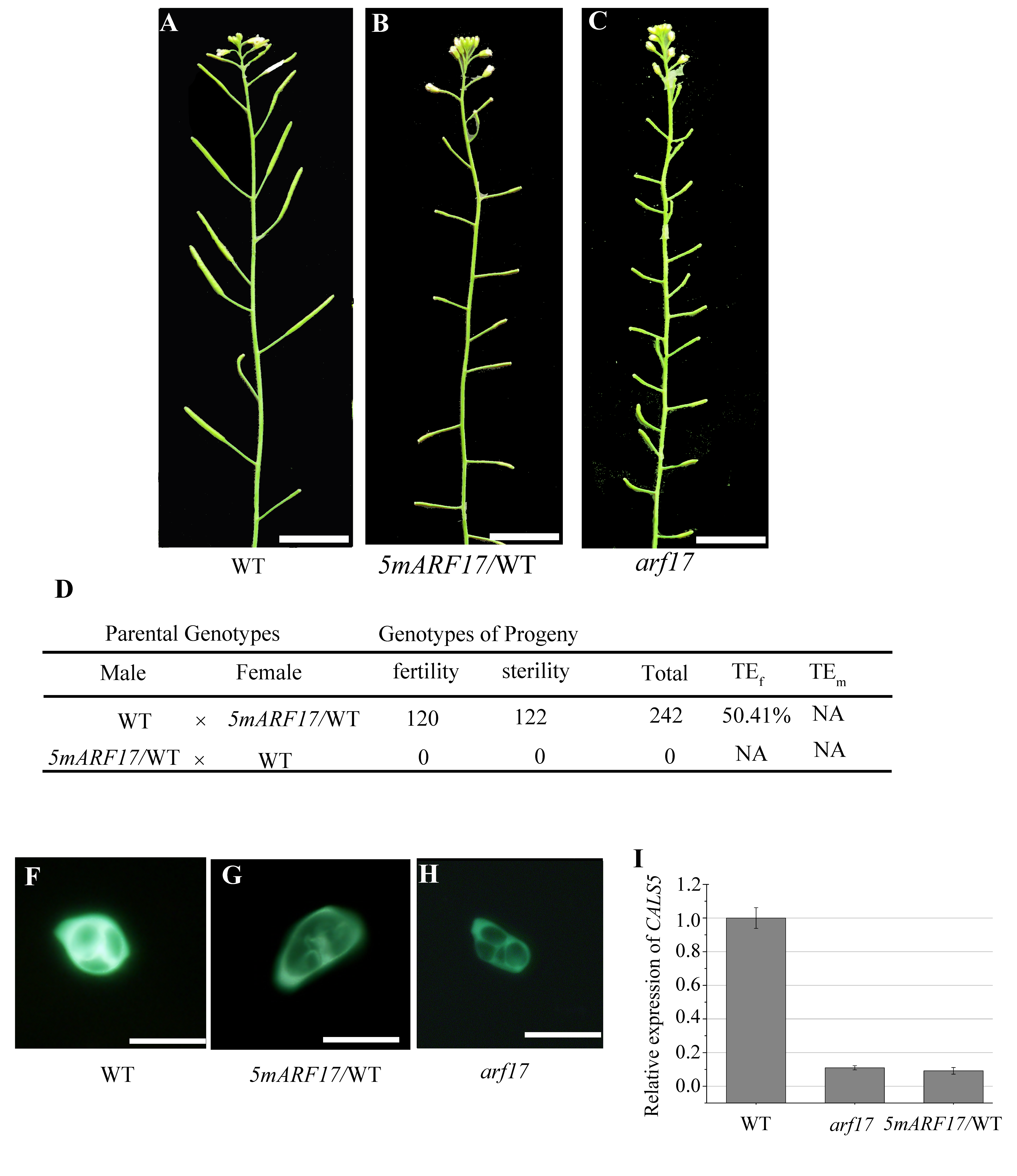

Supplement: Supplementary file 1 — The phenotype, segregation analysis and expression of CALS5 in 5mARF17/WT plants. A-C A 35-day-old wild-type plant (WT) (A); 5mARF17/WT plant (B) and arf17 mutant (C). Bars = 2 cm. (D) Transmission efficiency of 5mARF17/WT mutants. TEf: female transmission efficiency; TEm: male transmission efficiency; NA: no application. E-G Aniline blue staining of the callose from a WT plant (E); a 5mARF17/WT plant (F); and an arf17 mutant (G). Bars = 20 μm. (H) Quantitative RT-PCR analysis of CALS5 expression in WT, 5mARF17 and arf17 buds. The level of CALS5 was normalized to that of tubulin and compared with that of WT. Error bars indicate SD and were calculated from three biological replicates. (TIFF 5996 kb) [file 12870_2017_1185_MOESM1_ESM.tif]

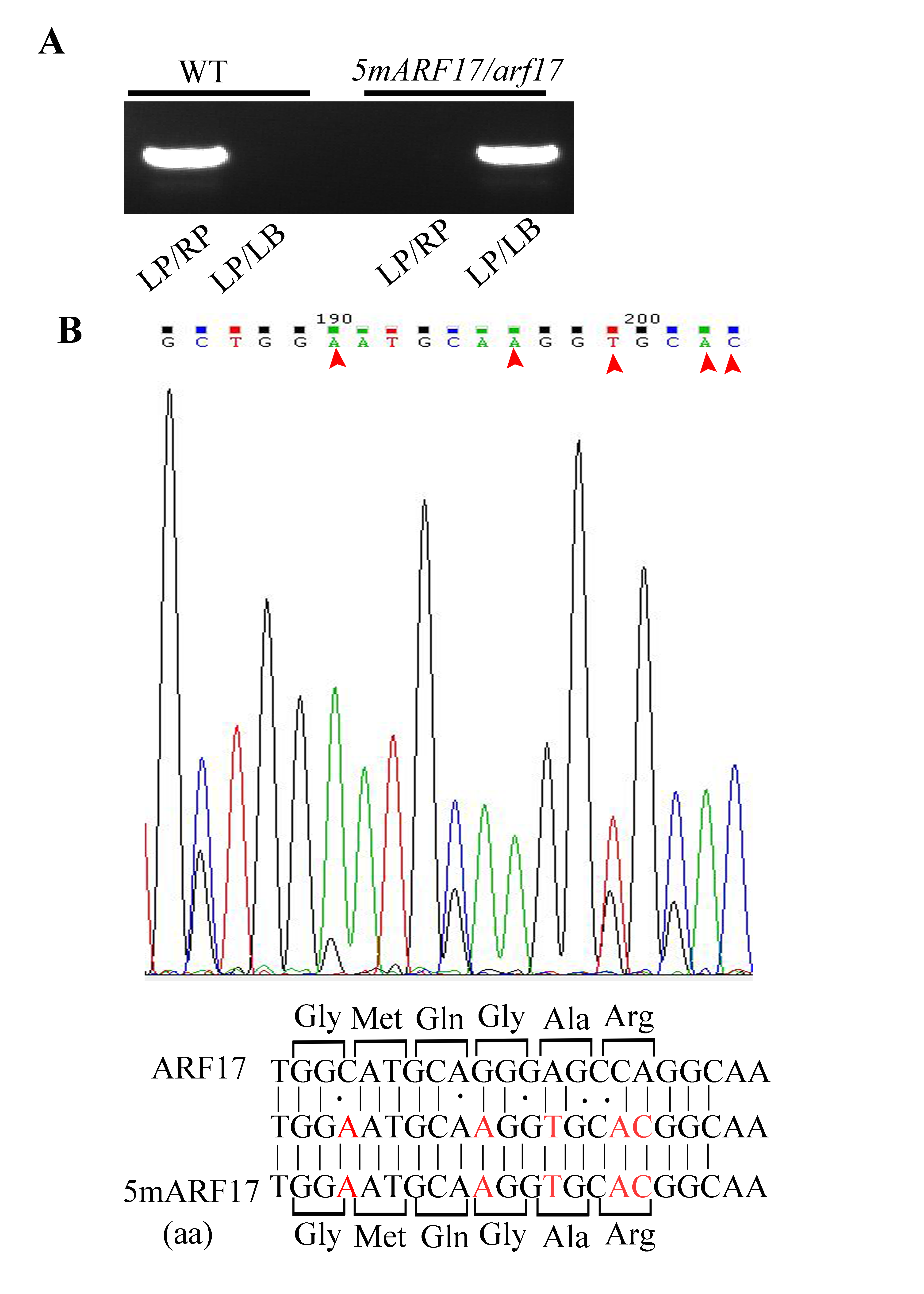

Supplement: Supplementary file 2 — Identification of sequences in a 5mARF17/arf17 plant. A Genomic PCR analysis of a 5mARF17/arf17 plant background. B Clone sequencing of 5 bases in 5mARF17/arf17 plants. (TIFF 4304 kb) [file 12870_2017_1185_MOESM2_ESM.tif]

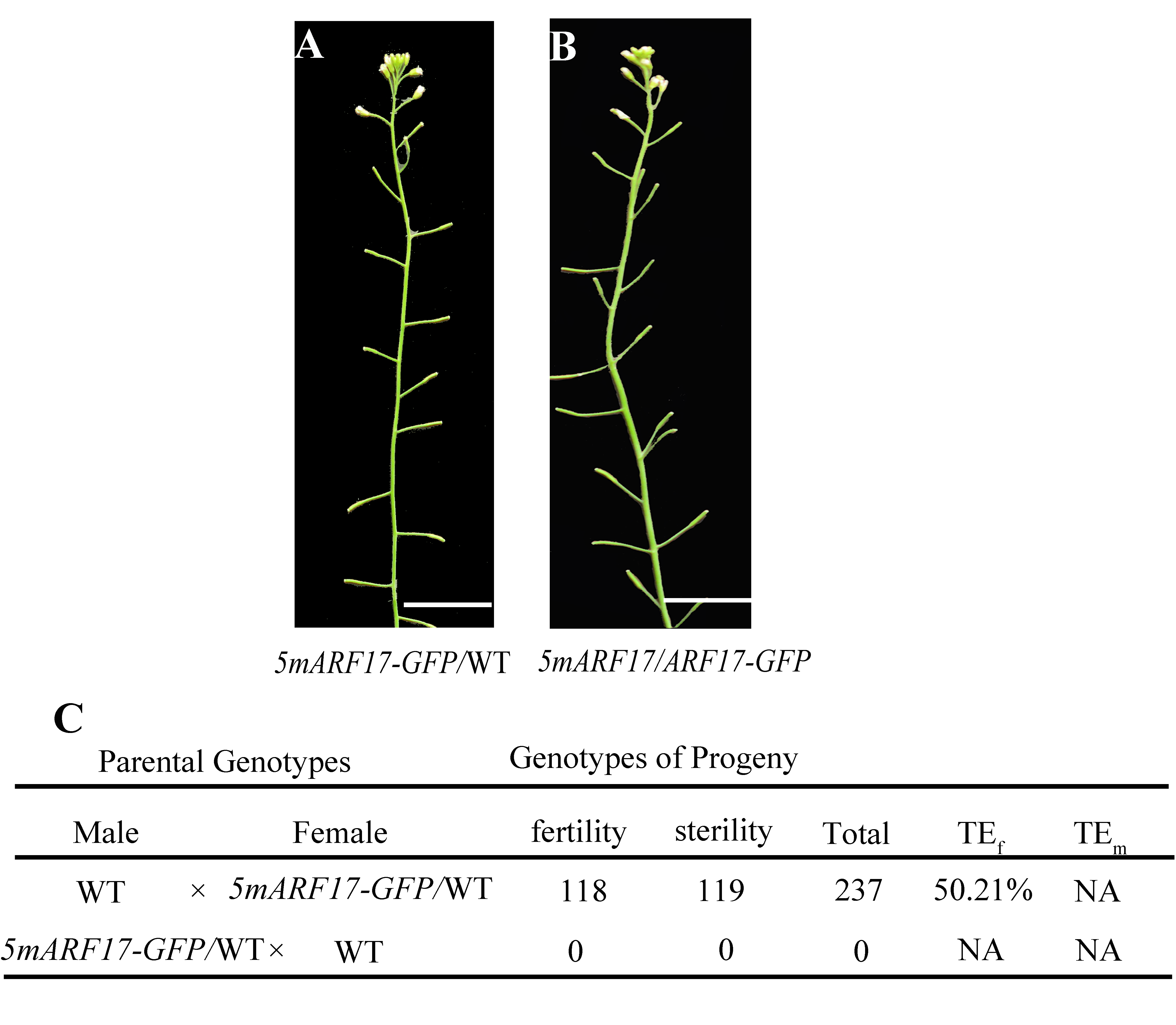

Supplement: Supplementary file 3 — Phenotype and segregation analyses of 5mARF17-GFP/WT plants. A and B A 35-day-old 5mARF17-GFP/WT plant (A) and ARF17-GFP/5mARF17 plant (B). Bars = 2 cm. (C) Transmission efficiency of a 5mARF17-GFP/WT plant. TEf: female transmission efficiency; TEm: male transmission efficiency; NA: no application. (TIFF 1132 kb) [file 12870_2017_1185_MOESM3_ESM.tif]

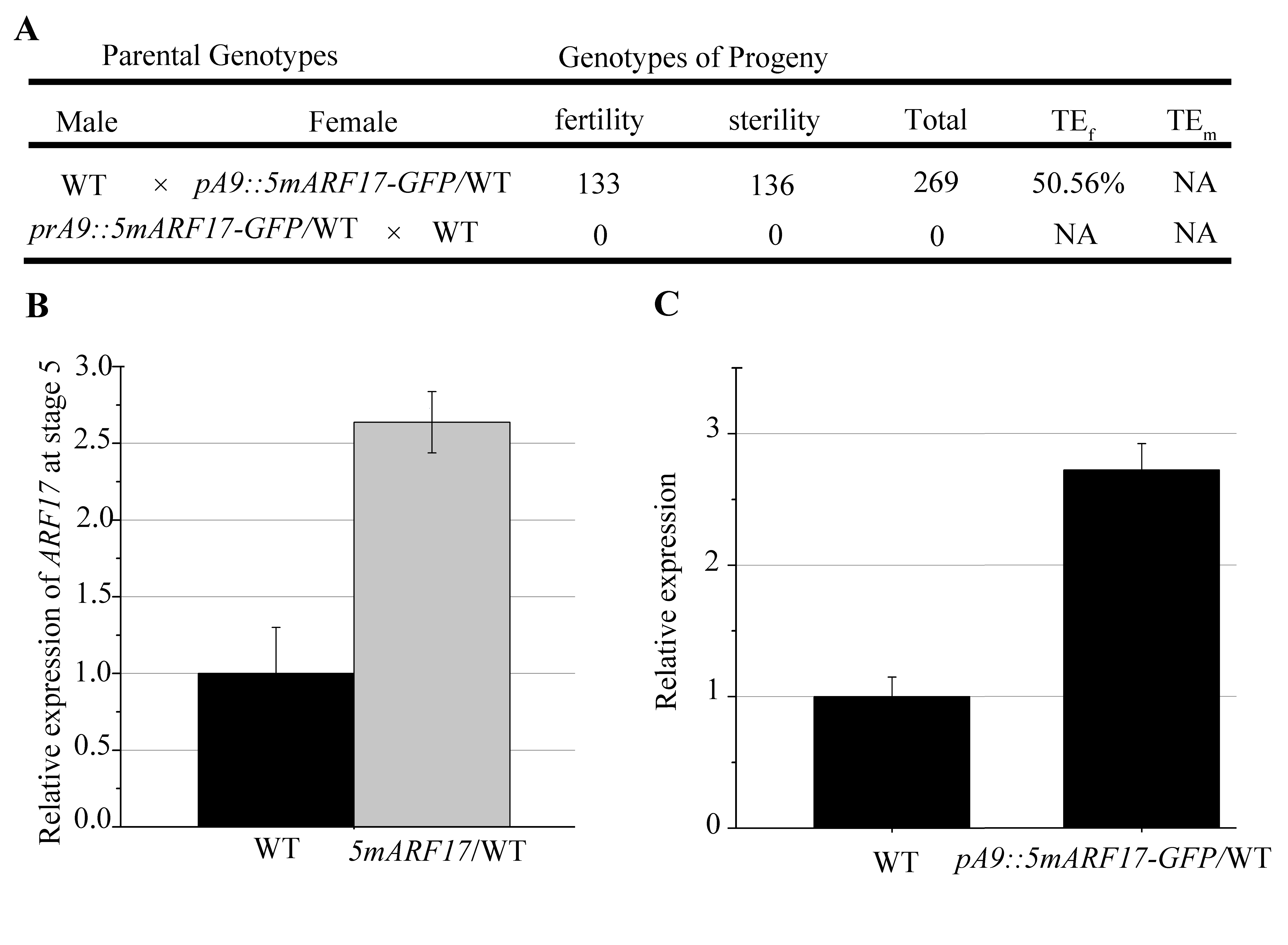

Supplement: Supplementary file 4 — Transmission efficiency of a pA9::5mARF17-GFP/WT plant. A Transmission efficiency of a promoterA9::5mARF17-GFP/WT (pA9::5mARF17-GFP/WT) plant. B Quantitative RT-PCR analysis of ARF17 expression in WT and 5mARF17/WT buds at stage 5. C Quantitative RT-PCR analysis of ARF17 expression in WT and pA9::5mARF17-GFP/WT buds. The level of ARF17 was normalized to that of tubulin and compared with that of WT. Error bars indicate SD and were calculated from three biological replicates. TEf: female transmission efficiency; TEm: male transmission efficiency; NA: no application. (TIFF 491 kb) [file 12870_2017_1185_MOESM4_ESM.tif]

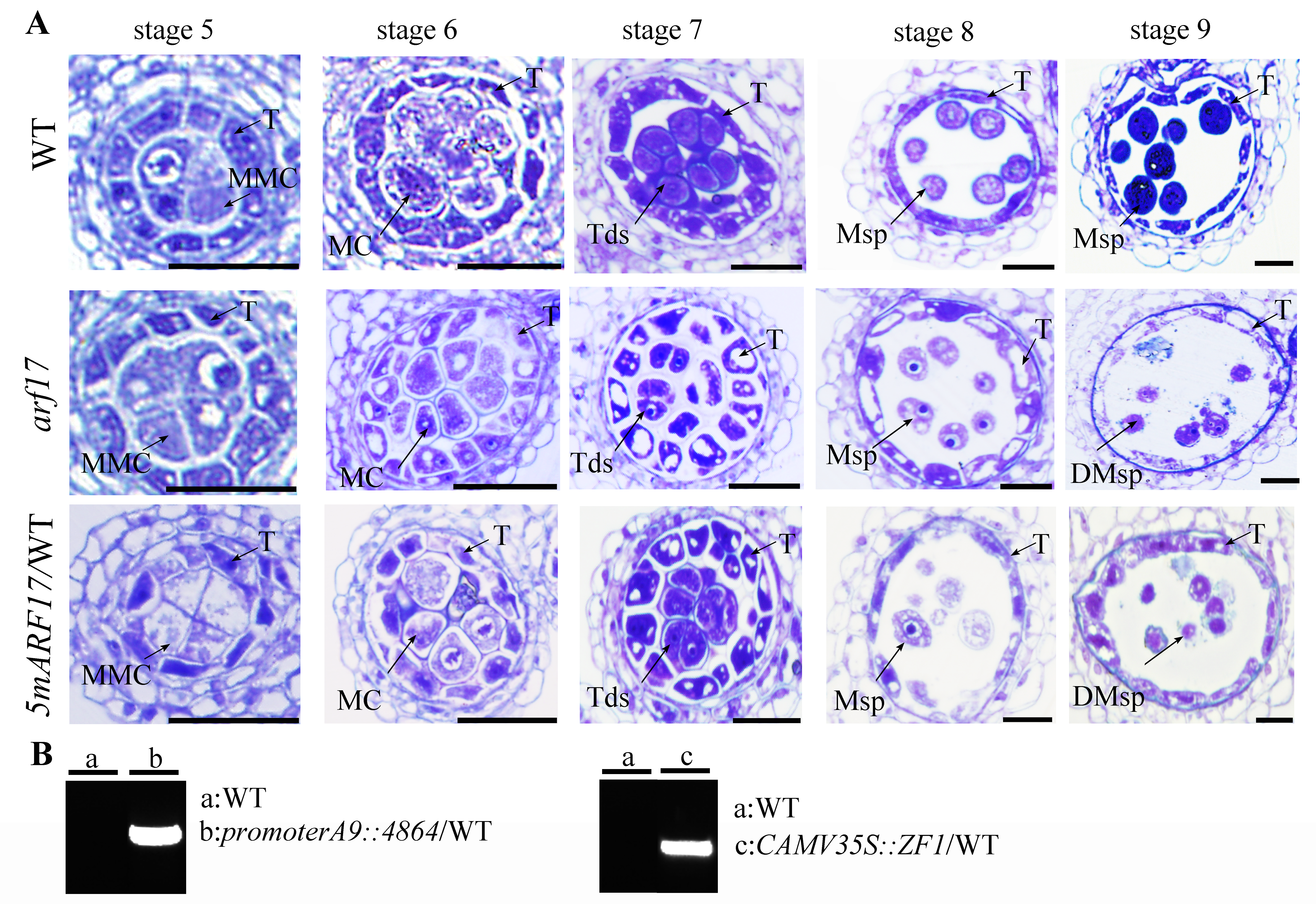

Supplement: Supplementary file 7 — Semi-thin sections of anthers and PCR analysis. A Semi-thin sections of anthers in WT, arf17 and 5mARF17/WT plants from stage 5 to 9. Bar = 20 μm. B Identification of transgenic sequences in WT, promoterA9:: AT1G48640/WT and CAMV35S::ZF1/WT. DMsp, degenerated microspore; MC, meiotic cell; MMC, microspore mother cell; Msp, microspore; T, tapetum; Tds, tetrads. (TIFF 23380 kb) [file 12870_2017_1185_MOESM7_ESM.tif]

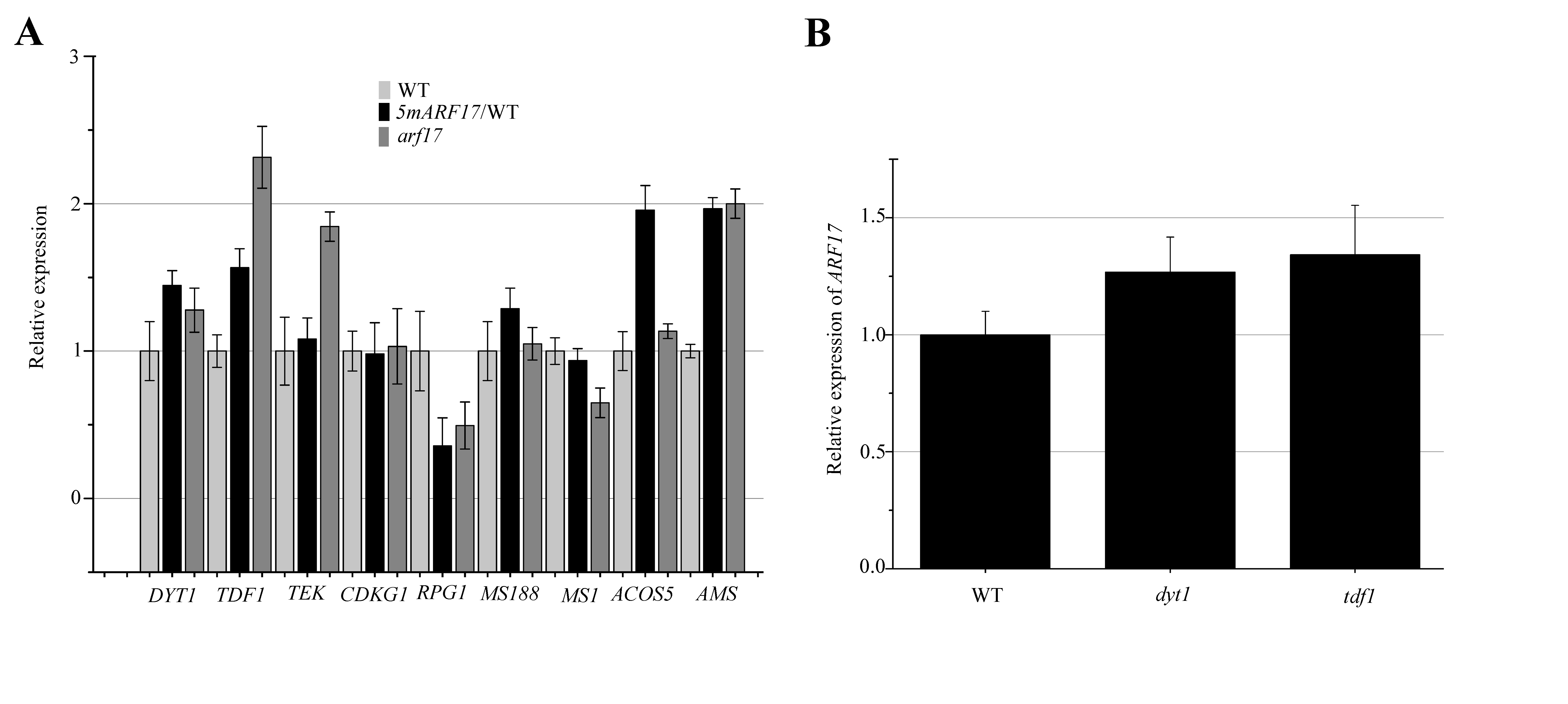

Supplement: Supplementary file 8 — Relative expression of tapetum- and pollen-formation genes in 5mARF17/WT and arf17 plants. A Quantitative RT-PCR analysis of the expression levels of genes involved in tapetum and pollen development. B Quantitative RT-PCR analysis of ARF17 expression in dyt1 and tdf1 mutants. The levels of DYT1, TDF1, TEK, CDKG1, RPG1, MS188, MS1, ACOS5, and ARF17 were normalized to those of tubulin and compared with those of WT. Error bars indicate SD and were calculated from three biological replicates. (TIFF 937 kb) [file 12870_2017_1185_MOESM8_ESM.tif]
